# Development of the interRAI home care frailty scale

**DOI:** 10.1186/s12877-016-0364-5

**Published:** 2016-11-21

**Authors:** John N. Morris, Elizabeth P. Howard, Knight R. Steel

**Affiliations:** 1 Quality of Care and Health-Care Standards Program, Institute for Aging Research, Hebrew SeniorLife, Boston, MA USA; 2 Northeastern University, Bouve College of Health Sciences, School of Nursing, Boston, MA USA; 3 Hackensack University Medical Center (emeritus), Hackensack, NJ USA

**Keywords:** Frailty scale, Home care, InterRAI, Assessment

## Abstract

**Background:**

The concept of frailty, a relative state of weakness reflecting multiple functional and health domains, continues to receive attention within the geriatrics field. It offers a summary of key personal characteristics, providing perspective on an individual’s life course.

There have been multiple attempts to measure frailty, some focusing on physiologic losses, others on specific diseases, disabilities or health deficits. Recently, multidimensional approaches to measuring frailty have included cognition, mood and social components. The purpose of this project was to develop and evaluate a Home Care Frailty Scale and provide a grounded basis for assessing a person’s risk for decline that included functional and cognitive health, social deficits and troubling diagnostic and clinical conditions.

**Methods:**

A secondary analysis design was used to develop the Home Care Frailty Scale. The data set consisted of client level home care data from service agencies around the world. The baseline sample included 967,865 assessments while the 6-month follow-up sample of persons still being served by the home care agencies consisted of 464,788 assessments. A pool of 70 candidate independent variables were screened for possible inclusion and 16 problem outcomes referencing accumulating declines and clinical complications served as the dependent variables. Multiple regression techniques were used to analyze the data.

**Results:**

The resulting Home Care Frailty Scale consisted of a final set of 29 items. The items fall across 6 categories of function, movement, cognition and communication, social life, nutrition, and clinical symptoms. The prevalence of the items ranged from a high of 87% for persons requiring help with meal preparation to 3.7% for persons who have experienced a recent decline in the amount of food eaten.

**Conclusions:**

The interRAI Home Care Frailty Scale is based on a strong conceptual foundation and in our analysis, performed as expected. Given the use of the interRAI Home Care Assessment System in multiple, diverse countries, the Home Care Frailty Scale will have wide applicability to support program planning and policy decision-making impacting home care clients and their formal and informal caregivers throughout the world.

## Background

Within geriatrics, the concept of frailty has attracted wide attention as the need to effectively utilize health resources for an expanding older adult population worldwide continues to grow [1, 2]. Frailty may be seen as a conceptual approach for bringing together personal characteristics within a summary measure that has a substantive bearing on a person’s life course [3]. It is often regarded as a description of individuals who are at risk for poor health outcomes [4, 5]. In our view, frailty is a relative state of weakness, with an expected gradual increase in the likelihood of future loss [6, 7]. Central to this concept is the idea that frailty incorporates multiple functional and health domains [8]. For the typical person, we are not speaking about situational losses with an expectation of full recovery from one or at most two health problems. Rather, in our view, a frailty assessment considers the full spectrum from a limited number of persistent problems to a true state of relative disability.

Currently, there are several scales and approaches to measuring frailty used in clinical practice and reported in the literature [9–14]. They include formal data collection tools as well as indirect sources of information. The PRISMA-7 questionnaire is designed to assess frailty via a telephone interview [11]. Distributed through the postal service or applied in practice by physicians, the 15-item Groningen Frailty Indicator includes the domains of physical, cognitive, social and psychological functioning [10]. Others have relied on an assessment of polypharmacy, the clinical judgment of the physician, or self-rated health status by the patient [12–14].

Some investigators have attempted to understand the underlying physiologic factors that might explain why frailty states make sense [1, 6, 15]. From this perspective, an appropriate scale or index of frailty would be based on a limited number of key markers of physiologic loss. Fried and her colleagues [6] created a hierarchical frailty index based on the sum of the person’s score on a limited number of key physiologic relevant dimensions. In this work, they included measures of muscle weakness, walking speed, weight loss, exhaustion, and low activity levels. These are key latent areas in any frailty scale construction effort, although the physical manifestation of these areas will vary. Further study revealed weight loss had a limited contribution while slow walking speed and a low level of physical activity had a strong relationship with the frailty index [16]. Other investigators further focused on outcomes and found the expected relationship over a 6 year period with respect to falls, mobility, activities of daily living, hospitalization, and death [5].

An alternative approach to creating a frailty measurement tool is represented in the work of Rockwood and his colleagues in which large numbers of health deficits are identified and then summed within a complex scale [17, 18]. We find this approach to be compelling, and in this paper we brought together a diverse series of latent concepts from within the interRAI Home Care assessment tool that could lead to heightened vulnerability [4, 18–20]. This type of multidimensional, accumulated deficit approach to frailty scale construction can thus incorporate physical, cognitive, clinical, and psycho-social components of frailty [1, 21, 22]. This paper describes one such measure – the interRAI Home Care Frailty Scale. It was derived from a subset of items in the widely used interRAI Home Care (interRAI-HC) assessment instrument [23] and provides a grounded basis for assessing the person’s risk of decline in a wide variety of areas. The interRAI-HC was designed to provide a comprehensive view of a population of persons with a variety of deficits and includes measures of cognition, communication, function, mood, behavior, social isolation, incontinence, health diagnoses, and clinical conditions, and services used [23]. The frailty literature has focused on just such concepts, and we integrated these items drawn from a comprehensive geriatric assessment into a new frailty scale. Our inclusion of possible items was quite broad. Functional measures included ADLs, IADLs, gait disorders, disability measures, and impairment measures that relate to the World Health Organization’s International Classification of Functioning [24–26]. Other areas of function included measures of cognitive performance, memory, and communication disorders [12, 27–29]. Physical related parameters included sensory loss, hearing loss, communication deficits, pain and other chronic clinical complications [27, 30, 31]. Diseases considered include the person’s cardiopulmonary and musculoskeletal systems [32, 33]. Contextual factors have referenced social despair, isolation, and mood disorders [34, 35].

## Methods

### Design and sample

A secondary analysis design was used to develop the Home Care Frailty Scale. The data set used in this paper consisted of client level interRAI Home Care data from service agencies around the world. Data were collected from the countries of Australia, Belgium, Canada, China, Finland, Germany, Iceland, Italy, Japan, Netherlands, New Zealand, Sweden, United Kingdom, and the United States. The client data came primarily from Canada (69% of baseline assessments) and the US (16% of baseline assessments). In Canada, the data represent all home care clients in the Provinces of Ontario and Manitoba, and a large sample of home care client sites from Nova Scotia. The home care data in the United States came mainly from all state supported home care clients in Massachusetts, Michigan, and Georgia. The baseline sample included 967,865 assessments, while the 6-month follow-up sample of persons still being served by the home care agencies consisted of 464,788 assessments.

In creating this scale, we followed the recommendations of Searle and colleagues [4] in selecting the items to be included in the scale. Specifically, we focused only on those independent variables that were related to a broad array of outcome characteristics – e.g., decline in function, cognitive decline. In this scale construction paradigm, the selected independent variables had to be shown to be associated with a summary scale that brought together troubling markers of decline. These measures had to represent conditions that could be expected to worsen with age, although concomitantly, they had to be conditions for which inter-person variations in the rates of change could be expected. Finally, the frailty scale had to incorporate items that covered a wide range of systems, including measures of cognition, functional performance, health status, social status, and clinical problems.

### Identifying independent variables

This task identified the full pool of over 70 candidate independent variables that were screened for possible inclusion in the interRAI Home Care Frailty Scale. The functional candidate items included a full panel of IADL and ADL measures, as well as several movement related items. The cognition and communication items included measures of memory, decision-making, management of finances, dementia, hearing, expressive communication, and receptive communication. The mental status items included measures of depression, anxiety, anhedonia, wandering, abuse, delusions, and hallucinations. The social items included measures of loneliness and social engagement. Nutrition items referenced weight loss as well as food and liquid consumption. The physical status items included measures related to pain, bone health, heart failure, respiratory status, cancer, renal failure, diabetes, stroke, dizziness, edema, head trauma, oral problems, vomiting, diarrhea, falls, and skin conditions.

### Adverse health outcomes, the dependent variables

Scientists working in the area of have examined diverse sets of outcome measures, including, falls, hospitalization, death, institutionalization, functional loss, and cognitive loss [6, 18, 30, 36–38]. With our efforts described here, we report on an extended outcome set of 16 measures. They reference functional loss, cognitive and communication decline, clinical instability, and heavy care service use. The set of 16 problematic outcomes reference the accumulating declines and clinical complications that can be expected to become more prevalent as one’s frailty score increases. Table 1 describes these functional, cognitive, clinical prognosis, and service measures. Each measure is scored as either a zero (0), for the condition not being present, or as a one (1) for the condition being present.

**Table 1:** Key concepts and dependency, dependent variables

Concept	Measure
Functional Decline	ADL status worse as compared to 90 days ago
	Overall self sufficiency has deteriorated as compared to 90 days ago
	In a typical over last 30 days the person did not leave the house
Cognition/Communication	Worsening decision making as compared to status 90 days ago
	Worsening communication (making self understood or understands others) as compared to status 90 days ago
Clinical Prognosis	Judged to have poor prospects of recovery from current disease or condition, improved health status expected
	Has conditions or diseases that make cognition, ADL, mood, or behavior patterns unstable (fluctuations, precarious, or deteriorating)
	Experiencing a flare-up of a recurrent or chronic problem
	Near end of life: Prognosis of less than 6 months to live or in hospice or receiving respite care
	Shortness of breath
	Self reported poor health
	Presence of a pressure ulcer
Service Use	Admitted to hospital for overnight stay in last 90 days
	Emergent care – including unscheduled nursing, physician, or therapeutic visits to office or home
	Daily nurse monitoring over last 7 days
	Physician or clinic visit over last 7 days

These measures were used in two ways for this effort. First, they were summed at baseline and the resulting sum was used as the dependent measure in an ordinary least squares regression equation to identify the independent variables that best entered the interRAI Home Care Frailty Scale. Second, the baseline and follow-up summed dependent variable scales, as well as selected subset of the individual measures (at baseline and follow-up) were displayed against the interRAI Frailty Scale scores. These individual selected measures included: worsening decision making, declining ADL status, self-reported poor health, and near end of life.

We also looked at how the frailty scale scores were related to the average hours of informal and formal supports received by the person. Here we looked at hours of care at baseline and follow-up, as well as assessments of the resiliency of the informal support provided.

### Analytical strategies

The data used here were provided pursuant to an agreement with interRAI to make use of its accumulated, cross-national home care data holdings to do research of this type. The analyses were covered by an approval from the Hebrew Senior Life, Institute for Aging Research, Institutional Review Board, and the analyses were completed using SPSS version 20.0.

We first evaluated all independent variables to identify those with a minimum correlation of 0.10 with the baseline sum of the 16 dependent measures. Next, these variables were subjected to regression analysis to identify those that made a unique contribution to the summary outcome measure. These measures then were summed to create the interRAI HC Frailty Scale. The internal consistency of the correlation among these items was assessed using the KR 20 alpha reliability estimate. The interRAI Frailty Scale was next assessed against a variety of dependent variables clusters, from the total count of dependent outcomes to a selected set of representative outcomes that made up the dependent summary scale. These assessments provided evidence of criterion-related validity.

## Results

Of the sample population at baseline, 60.4% were female and 36% were married. The median age of the sample population was 79 years with an interquartile range of 16. There was a linear relationship between the frailty index and chronological age (Pearson correlation = 0.10, non-linear Eta correlation = 0.11). As age increased, there was slight tendency for frailty scale score to increase. Nearly one-half (48.5%) had no ADL deficits, 3.1% had no IADL deficits, and 27% were fully dependent in IADLs. The cognitive performance scale, a cognitive measure within the interRAI Home Care assessment system demonstrated that 38.9% of the sample were cognitively intact or independent in all elements of cognition. Within this sample, 60.2% had no symptoms of depression and 25.5% had 2 or more depressive symptoms.

The regression of all independent items with a minimum of 0.10 correlation with the dependent variable count measure resulted in a final 29 variable frailty risk set. Table 2 lists these measures. The items fall across 6 categories and include function, movement, cognition and communication, social life, nutrition, and clinical symptoms. The prevalence of the items range from a high of 87% for persons requiring help in meal preparation to 3.7% for persons who have had experienced a recent decline in the amount of food eaten.

**Table 2:** interRAI home care frailty scale items and associated correlations

Variable	Definition (Code of “1” is added)	% With condition	Mean frailty score among those with condition (Mean = 9.4)	Corr with summed dep var at baseline	Corr with Summed dep var at follow-up	Corr with interRAI frailty scale
Function						
IADL – Housework	Ext Assistance	71.6	11.2	0.31	0.16	0.58
IADL – Meals	Ext Assistance	58.2	12.3	0.33	0.18	0.67
IADL - Meals	Any Problem	86.8	10.4	0.28	0.16	0.50
IADL – Phone Use	Any Problem	21.0	14.8	0.22	0.11	0.55
ADL – Personal Hygiene	Any Problem	45.3	13.2	0.35	0.17	0.67
ADL – Locomotion	Physical Help	18.5	15.8	0.33	0.14	0.59
ADL - Transfer	Extensive Help	20.8	15.2	0.31	0.12	0.58
ADL – Toilet Use	Any Problem	30.6	14.0	0.30	0.13	0.60
Movement or Movement Related						
Climb Stairs	Not Indep	69.6	11.0	0.30	0.13	0.50
Hrs of Phy Activity	<2 h in 3 days	47.6	11.9	0.27	0.15	0.47
Fell in Last 90 Days	Yes	15.1	11.8	0.16	0.11	0.21
Dizzy	Yes	18.0	10.6	0.13	0.10	0.12
Cognition and Communication						
Cog – Decision Making	Not Indep	47.5	12.2	0.25	0.18	0.53
IADL - Manage Medication	Ext Assistance	36.2	13.6	0.27	0.16	0.61
IADL – Manage Finances	Any Problem	70.5	11.3	0.25	0.15	0.57
Dementia Other Than Alzhimers	Yes	16.2	13.1	0.15	0.11	0.34
Understand Others	Not Indep	30.5	13.1	0.23	0..16	0.48
Social						
Decline in Soc Act	Yes -- (if yes, count of “2” rather than “1”)	43.0	11.7	0.36	0.18	0.39
Reduced Soc Act	Yes	14.1	12.4	0.19	0.13	0.25
Withdrawal From Activities of Interest	Yes	4.9	14.3	0.15	0.10	0.20
Nutritional Status						
Weight Loss	Yes	11.1	12.5	0.22	0.10	0.21
Loss of Appetite	Yes	11.0	12.4	0.21	0.12	0.21
Decrease in Food Eaten	Yes	3.7	14.3	0.16	0.06	0.19
Clinical Symptoms and Diagnoses						
Bowel Incontinent	Some +	19.4	14.5	0.25	0.12	0.49
Urinary Tract Infect	Yes	6.9	12.7	0.15	0.06	0.20
Renal Failure	Yes	14.2	12.4	0.12	0.06	0.18
Pneumonia	Yes	7.7	13.7	0.14	0.06	0.18
Conges Heart Fail	Yes	15.2	11.4	0.16	0.11	0.19
Emphysema	Yes	17.9	10.3	0.16	0.12	0.11

**Fig. 1:**
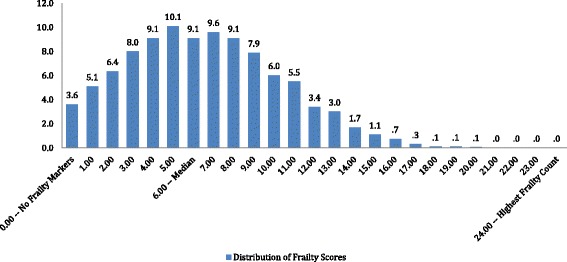
interRAI home care frailty scale (Mean = 6.56, Median = 6, sd = 3.76, N = 964,479) (KR 20 Alpha Reliability = 0.75)

Figure 1 displays the distribution of the interRAI Home Care Frailty Scale at the baseline assessment. In this cross-national home care population, 96% of persons have one or more of the problem factors that make up the scale. The mean score was 6.6, the median score was 6, and there was a progressive decrease in persons in the scale categories as one moved beyond the median to the highest score of 24. Scale scores 15 through 23 included approximately 3% of the home care clients.

**Fig. 2:**
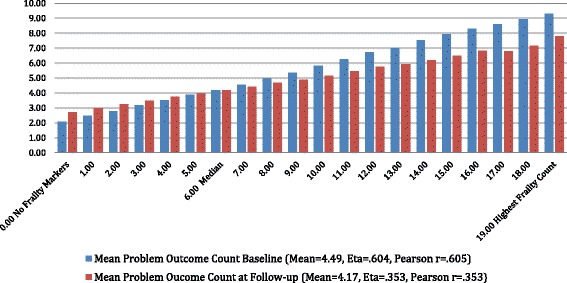
Mean problem outcome count vs. interRAI HC frailty scale

Figure 2 displays the cross-walk between the count of problem outcomes (which ranged from 0 to 16 and the baseline interRAI Home Care (HC) Frailty Scale (with scores of 19 or higher rounded to 19). This figure displays results at the baseline and 6-month follow-ups. At both time points average number of problematic outcomes rises in a linear fashion across the categories of the interRAI HC Frailty Scale. In this cross-national home care population, persons with the best score on the interRAI HC Frailty Scale averaged about 2.1 to 2.7 problematic outcomes. At the median point on the interRAI HC Frailty Scale, this count had risen to about 4.2. At the highest (worst) category of the interRAI HC Frailty Scale the problematic outcome mean rose to 9.3 at baseline and 7.8 at follow-up.

**Fig. 3:**
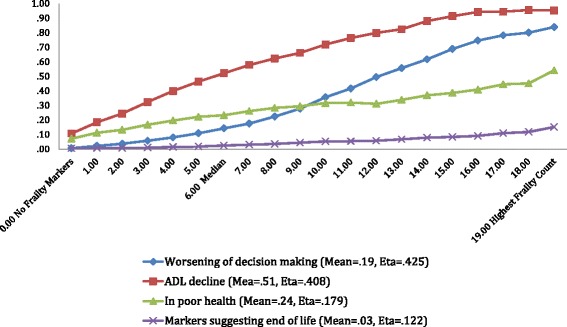
interRAI HC frailty scale and incidence of 4 problematic outcomes

Figure 3 displays the baseline-and follow-up scores for four of the items in the problematic condition count: cognitive decline, functional decline, in poor health, and near the end of life indicators. The prevalence for each dependent measure rises across the increasing score count for the interRAI HC Frailty Scale. The slope is greatest for the cognitive and functional decline measures, and least for the measure that indicates that the person is near the end of life.

**Fig. 4:**
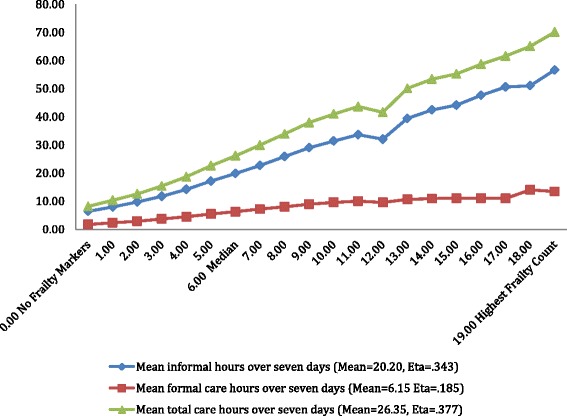
interRAI frailty scale and weekly mean hours of care

Figure 4 displays a measure of personal dependency that is outside the dependent variable count used above, referencing the hours of informal, formal and total care the person received across the values of the interRAI HC Frailty Scale. The level of informal help received from family and friends during the week rises steadily from 6 h a week for those with no frailty risk markers, to 20 h a week at the median point, and to 57 h a week for persons with a score of 19 or higher. The increase in formal care hours is more muted, rising from 1.7 to 13.4 h of care per week.

**Fig. 5:**
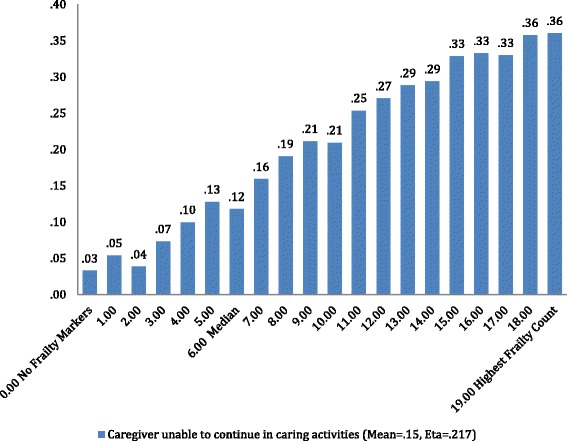
interRAI frailty scale and proportion of informal caregivers unable to continue in caring activities

Figure 5 looks at informal caring activities in yet another dimension, displaying the proportion of persons for whom there is a concern about their ability to continue in their caring role. Here that rate begins at 3% for persons with no frailty markers, to 12% at the median point, and 36% for those with 19 or more frailty markers.

## Discussion

We have presented the development and evaluation of the interRAI HC Frailty Scale that is based on assessment items within the interRAI Home Care Assessment System. As such, the HC Frailty Scale has emerged from a comprehensive geriatric assessment, in contrast to a recommendation that identification of frailty be followed by a comprehensive assessment [39]. Here, the frailty may be assessed and reassessed at scheduled intervals to obtain a scale score in addition to a repeated comprehensive evaluation without additional time or resources.

Using a cross-national data set of 967,865 baseline assessments and 464,788 6-month follow-up assessments, 70 variables were independently screened for inclusion in the Frailty Scale. The final scale consisted of 29 assessment items that best correlated with a select group of dependent measures representing accumulating declines and clinical complications. The frailty scale items address the areas of function, movement, cognition and communication, social life, nutrition, and clinical symptoms. The resulting scale is consistent with prior work demonstrating frailty as a relative state of weakness with expectant future loss [6, 7]. The positive relationship between frailty score and chronological age was present, similar to other studies but there is strong evidence of the multi-dimension components of frailty [40, 41].

The frailty scale scores extended from zero or no frailty markers to a high of 29. Approximately 3% of the home care clients had frailty scores between 15 and 23 indicating that with a high level of frailty, an individual would be less likely to remain at home. Conversely, the distribution of frailty scores clustered towards the lower end of the scale as one might expect given the overall health status of the sample was stable enough to reside in the community and receive support at home. Evidence of criterion-related validity was reflected in the comparison of frailty scores with proportion of home care clients experiencing problematic outcomes of cognitive decline, functional decline and self-reported poor health. Markers suggesting end of life, although related to increasing frailty, rose at a significantly lower rate than did the other outcomes. The relationship between the frailty scores and weekly hours of care required further validates the measure. Notably, weekly formal care hours gradually increase with higher frailty scores. In contrast, the weekly informal care hours increase sharply with rising frailty scores. This outcome call attention to the need to further examine the roles and responsibilities of the informal caregiver as well as the support available to assist these often unacknowledged and ‘unofficial’ health care providers. The increasing proportion of informal caregivers reporting an inability to continue with care activities provides a further imperative to address the needs of this group.

The approach in developing the Frailty Scale from items contained in the interRAI Home Care Assessment tool is similar to the frailty index developed from data gathered at a geriatric day-hospital unit in Toulouse frailty clinic [16]. The items from this scale included chronic diseases, basic and instrumental disabilities, serum Vitamin D, cognition, physical performance, obesity, visual and hearing impairment and malnutrition. In comparision, the interRAI Home Care Frailty Scale contains items representing physical function, movement, cognition and communication, nutritional status, and clinical symptoms and diagnoses.

In creating a frailty measurement tool Rockwood and his colleagues [17, 18] had a compelling approach in which large numbers of health deficits are identified and then summed within a complex scale. With our efforts we brought together a diverse series of latent concepts from within the interRAI Home Care assessment tool that could lead to heightened vulnerability [4, 18–20]. This type of multidimensional, accumulated deficit approach to frailty scale construction thus incorporated physical, cognitive, clinical, and psycho-social components of frailty [1, 21, 22].

## Conclusion

The interRAI Home Care Frailty Scale provides a summary measure of personal characteristics impacting an individual’s life course. The scale is based on a strong conceptual foundation and in our evaluation, performed as expected. Items for the Home Care Frailty Scale originate from the interRAI Home Care assessment system which is a comprehensive geriatric assessment completed on home care clients at pre-specified intervals. It is used across the globe including such diverse countries as the US, Canada, New Zealand, Hong Kong, Finland, Italy, and France. Thus, this new frailty scale will thus have wide applicability. There is a wide score range and a diverse set of outcome measures have now been shown to track with this scale.

Prior work has demonstrated that frailty, at earlier stages, may be reversible [42, 43]. Early and consistent measurement of frailty are key to interventions that may prevent decline and increased dependency among older adults. The interRAI Home Care Frailty Scale is well positioned to work in such a way, impacting the home care population in multiple nations throughout the world without the need for additional data collection tools, time or resources.

This scale also may serve as a valuable instrument for program planning and policy decision-making impacting home care clients and their formal and informal caregivers throughout the world.
